# Urinary pyridinoline cross-links as biomarkers of osteogenesis imperfecta

**DOI:** 10.1186/s13023-015-0315-9

**Published:** 2015-08-27

**Authors:** Uschi Lindert, Marius Kraenzlin, Ana Belinda Campos-Xavier, Matthias R. Baumgartner, Luisa Bonafé, Cecilia Giunta, Marianne Rohrbach

**Affiliations:** Division of Metabolism, Connective Tissue Unit, University Children’s Hospital Zurich and Children’s Research Centre, Zurich, Switzerland; Clinic for Endocrinology, Diabetology and Metabolism, University Hospital, Basel, Switzerland; Center for Molecular Diseases, Lausanne University Hospital, Lausanne, Switzerland

**Keywords:** Osteogenesis imperfecta, Biomarker, Urinary pyridinoline cross-links, LP/HP ratio, Collagen, Mutations, Non-accidental injury

## Abstract

Osteogenesis imperfecta (OI) is a group of genetic heterogeneous connective tissue disorders characterized by increased bone fragility and susceptibility to fractures. Laboratory diagnosis relies on time-consuming and cost-intensive biochemical and molecular genetics analyses. Therefore, it is desirable to identify and establish new diagnostic markers for OI that are reliable, cost-effective and easily accessible. In our study we have identified the ratio of the urinary pyridinoline cross-links lysyl-pyridinoline and hydroxylysyl-pyridinoline as a promising, time- and cost-effective biomarker for osteogenesis imperfecta, that could be used furthermore to investigate cases of suspected non-accidental injury in infants.

## Dear Editor,

Osteogenesis imperfecta or “brittle bone disease” (OI) is a clinically and genetically heterogeneous disorder of bone matrix formation and remodelling, presenting with low bone mass, bone fragility and deformity, short stature, grey or blue sclera, and hearing loss. Its prevalence is about 1 in 15,000 to 20,000 births [1]. Approximately 90 % of OI cases are caused by dominant mutations in *COL1A1* and *COL1A2* resulting in quantitative and/or qualitative alterations of type I collagen, the major extracellular matrix component of bone and skin. A proportion of the remaining 10 % is due to either dominant or recessive mutations in several noncollagenous genes involved in the post-translational processing of procollagen I, in intracellular collagen transport, in osteoblast-specific signaling or in gene regulation [2].

Diagnosis of OI and identification of the disease-causing gene is important i) to end the patient’s quest for the disease causing his or her symptoms; ii) to allow for prenatal diagnosis, iii) to clarify cases of suspected “non-accidental injury” or “battered child syndrome”; iv) and to investigate the underlying pathomechanism in order to develop new therapeutic approaches. Establishing a specific diagnosis currently relies on biochemical analysis of collagens secreted by cultured fibroblast obtained by skin biopsies, and on molecular genetic analysis of the known OI related genes. The accessibility of modern genomic sequencing technologies has reduced the cost of molecular genetic testing, which was almost prohibitive in the past, due to the large number of genes, and exons per gene, to be sequenced. However, the interpretation of the molecular testing often requires biochemical investigations aimed to infer pathogenecity to the identified sequence variant if it is not yet reported on specialized mutation data bases such as the Database of Osteogenesis imperfecta variants at http://www.le.ac.uk/genetics/collagen/. Therefore, the cost remains a hurdle in OI diagnostics.

The urinary cross-links lysyl-pyridinoline (LP, or deoxypyridinoline DPD) and hydroxylysyl-pyridinoline (HP, or pyridinoline PYD) are established biochemical markers of osteoclastic bone resorption and collagen degradation. Pyridinolines are formed during fibril formation of type I and type II collagen in the extracellular matrix. Some lysyl- and hydroxylysyl-residues in distinct positions in the collagen triple-helix and in the telopeptides of the tropocollagen are oxidised by lysyl oxidase. Subsequently three of these residues are covalently linked thereby interconnecting tropocollagen molecules and stabilizing the collagen matrix. When bone collagen is degraded, pyridinolines remain as stable degradation products and are secreted with the urine. Depending on whether or not these lysyl residues had been hydroxylated prior to crosslinking, lysyl-pyridinoline (LP) or hydroxylysyl-pyridinoline (HP) is generated. Although LP and HP values in the urine vary substantially depending on age and time of sampling, their ratio is remarkably constant (LP/HP: 0.2 + 0.03) in healthy individuals at any age [3]. Furthermore, LP/HP ratios are proven diagnostic tools for genetic disorders of collagen metabolism such as the kyphoscoliotic type of Ehlers-Danlos syndrome (EDS VIA) [4].

Since both dominant and recessive forms of OI lead to abnormal cross-linking chemistry in bone collagen [5], we hypothesize that the urinary LP/HP ratio may serve as a diagnostic biomarker for OI.

## Methodology

The study was conducted according to the declaration of Helsinki, and approved by SwissEthics. To test whether the ratio of urinary pyridinolines LP/HP may be used as a biomarker for OI, we measured LP and HP cross-links by automated HPLC [3] in spot urines of 49 genetically defined OI patients. The study cohort included patients with mutations in the following genes: *COL1A1* or *COL1A2* (*n* = 24; including 6 patients aged 0.1-24 months), *LEPRE1* (*n* = 8), *CRTAP* (*n* = 4), *SP7/OSX* (*n* = 1), *BMP1/mTLD* (*n* = 2), *SERPINF1* (*n* = 2), the recurrent c.-14C > T in *IFTM5* (*n* = 3), *WNT1* (*n* = 1), *PLOD2* (*n* = 1) and *FKBP10* (*n* = 3), as well as heterozygous carriers for mutations in *LEPRE1* (*n* = 2), *CRTAP* (*n* = 2) and *SP7/OSX* (*n* = 2).

## Findings

**Fig. 1:**
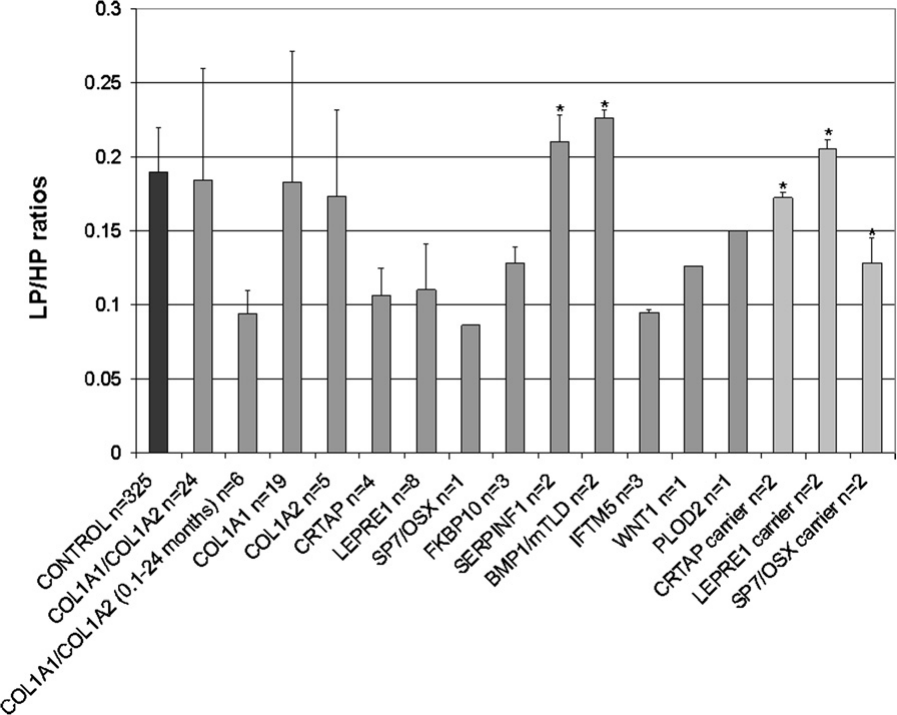
LP/HP ratios of OI patients, heterozygote carriers and controls [3]. OI patients with mutations in *LEPRE1*, *CRTAP*, *SP7/OSX*, *FKBP10*, *WNT1* and *IFTM5* show decreased LP/HP ratios compared to controls. Heterozygous carriers for a *SP7*/*OSX* mutation show slightly decreased ratios; carriers for *LEPRE1* and *CRTAP* mutations show normal LP/HP ratios. OI patients with mutations in *SERPINF1* or *BMP1/mTLD* have LP/HP ratios within the control range. In individuals with *COL1A1*/*COL1A2* mutations LP/HP ratios were normal in 15/24, decreased in 8/24 and increased in 1/24. 6 out of 8 patients presenting with low LP/HP ratios were aged 0.1-2 years. Error bars indicates standard deviations (SD) or the highest measured value * (sample size *n* = 2)

LP/HP ratios are shown in Fig. 1. Compared to control LP/HP ratios (0.20 ± 0.03, *n* = 325) [3], we found (i) markedly decreased LP/HP ratios in OI patients with mutations in *LEPRE1* (mean: 0.11), in *CRTAP* (mean: 0.106), in *SP7/OSX* (0.086) and in *IFTM5* (mean: 0.095); (ii) moderately decreased ratios in patients with mutations in *FKBP10* (mean: 0.128), in *WNT1* (0.126), as well as in heterozygous carriers for a mutation in *SP7/OSX* (mean: 0.128); (iii) normal LP/HP ratios in patients with mutations in *SERPINF1* (mean: 0.195), in *BMP1/mTLD* (mean: 0.226), and in heterozygous carriers for *LEPRE1* (mean: 0.2) and *CRTAP* (mean: 0.172) mutations. In patients with *COL1A1/COL1A2* mutations (mean: 0.184; *n* = 24), the LP/HP ratios were normal in 15/24, decreased in 8/24 and increased in 1/24. 6 out of 8 patients presenting with decreased LP/HP ratios were aged 0.1-2 years (normal values: 0.172 ± 0.02, age 1-12 months; 0.193 ± 0.03, age 1-3 years) [3].

Bisphosphonate treatment did not influence the ratio of total urinary pyridinoline cross-links (data not shown).

## Conclusions

These results identify the urinary pyridinoline ratio LP/HP as a promising biomarker for some forms of OI. In particular, it has the potential to detect recessive forms of OI caused by mutations in the genes *LEPRE1*, *CRTAP*, *FKBP10*, *WNT1* and *SP7/OSX*, and to discriminate between the dominant inherited forms caused by *COL1A1/COL1A2* and *IFTM5* mutations, therefore improving the efficacy of the final diagnosis and reducing the time and costs of laboratory investigations. Furthermore, the urinary LP/HP ratios might be helpful in investigating cases of suspected non-accidental injury in babies and infants aged 2 years and below.

More samples will be needed to establish urinary pyridinolines as a screening tool for OI diagnostics. Therefore, with this report we hope to attract more cases of OI with a known genetic defect in order to statistically validate our preliminary study.

## Abbreviations

DPD: deoxypyridinoline
HP: hydroxylysyl-pyridinolines
LP: lysyl-pyridinolines
OI: osteogenesis imperfecta
PYD: pyridinoline
